# Effect of counseling intervention on symptoms, pain acceptance and psychological wellbeing among patients with fibromyalgia

**DOI:** 10.1186/s12912-025-03333-0

**Published:** 2025-06-20

**Authors:** Ayman Muhammad Kamel Senosy, Hoda Sayed Mohamed Abd El Naby, Eman Ahmed Elhaweet, Basma ElAraby El-Feqi

**Affiliations:** 1 Medical Surgical Nursing Department, Faculty of Nursing, Ain Shams University, Cairo, Egypt; 2 Psychiatric and Mental Health Nursing Department, Faculty of Nursing, Ain Shams University, Cairo, Egypt

**Keywords:** Fibromyalgia, Pain acceptance, Psychological well-being, Symptoms

## Abstract

**Background:**

Nurses to assist patients make the best choices in challenging circumstances, counseling is crucial. Patients’ health and quality of life can be enhanced by healthcare providers, particularly nurses, through counseling and educational initiatives. Fibromyalgia is characterized by widespread pain that negatively impacts an individual’s ability to work. People may be able to improve their physical and mental health and accept suffering with the use of nursing counseling methods.

**Aim:**

This study aimed to investigate the effect of counselling intervention on symptoms, pain acceptance, and the psychological well-being of patients with fibromyalgia.

**Design:**

A quasi-experimental (study and control group pre/post-test) design included 120 patients with fibromyalgia during their follow-up visits to the outpatient rheumatology clinic at El-Demerdash Hospital affiliated with Ain Shams University Hospitals, Cairo, Egypt.

**Tools for data collection:**

**(1)** Structured Interview Questionnaire for Patients with Fibromyalgia, **(2)** Pain Acceptance Questionnaire, **(3)** Psychological well-being Questionnaire.

**Results:**

Post intervention, there were statistically significant differences between the groups in terms of psychological well-being, pain acceptance, and symptoms (*p* < 0.05). Regarding the total level of pain acceptance post implementation of the counseling intervention, there was a highly statistically significant difference between the study and control groups. Post the implementation of the counseling intervention, there was a statistically significant difference in the study group’s anxiety, depression, vitality, positive well-being, and general health (0.03*, 0.01**). Additionally, the study group’s pain willingness and activity engagement subscales showed statistically significant differences, with a p-value of less than 0.001. Additionally, there were statistically significant differences between the groups pre and post the program’s implementation in terms of psychological well-being, pain acceptance, and symptoms (*p* < 0.05).

**Conclusions:**

Patients with fibromyalgia under research report statistically significant improvements in their psychological well-being, pain acceptance, and symptoms when counseling interventions are used.

**Recommendations:**

In addition to the standard nursing care provided to patients in all fibromyalgia treatment clinics, counseling interventions must be included. Clinics must schedule counseling sessions on a monthly basis. To increase their understanding and boost their mental and physical health, it should be updated on a regular basis. More study is needed to evaluate the biopsychosocial needs of such people and treat all the fibromyalgia-related signs and symptoms.

**Clinical trial number:**

Not applicable.

## Background

Fibromyalgia is the second most common rheumatic disorder globally, and is considered of the most common chronic musculoskeletal pain conditions and. About 2–7% of the general population is affected by FM, with a higher prevalence in women. Fibromyalgia is a syndrome that features widespread pain all over the body [1]. People with fibromyalgia may be more sensitive to pain than people without fibromyalgia. Patients with fibromyalgia experience more pain (during rest or after movement) and more impairment in their overall functioning and impact than those with other chronic musculoskeletal pain [2].

Patients with fibromyalgia typically present with exhaustion, poor sleep, and chronic widespread pain, sometimes known as multisite pain. It is frequently associated with other symptoms such fatigue, headaches, digestive problems, joint and muscular stiffness, and sleeplessness [3]. People who have FM may not be taken seriously by others, who may downplay or ignore the severity and frequency of their symptoms, making it a “invisible” condition. Patients may believe that in order to defend themselves, they must explain or verify their symptoms. Patients with fibromyalgia may feel more depressed, anxious, and alone as a result of this [4].

There is a close relationship between fibromyalgia and mental and psychological wellness. According to experts, it has a significant detrimental impact on psychological well-being, including anxiety, despair, vigor, self-control, and overall health [5]. When fibromyalgia is diagnosed, depression is present in about 40% of individuals. About 60% of fibromyalgia patients will experience anxiety at some point in their lives, whereas 30% of cases will have anxiety disorder at the time of diagnosis (10–14). It appears that the degree of cognitive impairment in fibromyalgia patients is correlated with their anxiety and depression levels [3]. Patients with fibromyalgia report feeling less healthy and having a reduced quality of life, and they are less productive. Fibromyalgia and its symptoms may be caused and developed in large part by physical and psychological stress brought on by living with pain, as well as emotionally taxing life events that come from living with pain [6].

Therefore, by addressing people’s relationship to their internal experiences, pain acceptance, stress reduction, depression and anxiety management, and commitment therapy may ultimately result in increased self-control and an improvement in psychological and physical welfare [7]. In patients with fibromyalgia, more pain acceptance is linked to improved daily functional levels as well as decreased pain and other co-occurring symptoms. Patients who are better informed are better able to accept their suffering and engage in self-care practices [8].

Clinical research suggests that pharmacological treatment alone is not the best approach, and integrated counselling intervention on how to accept the pain and how to improve psychological well-being improves outcomes in these patients [5]. Considering the prevalence of FM and the substantial cost of care, it is important to develop and implement counselling intervention programs designed specifically for people with FM to help them accept the pain and improve their psychological and physical well-being [9].

### Significance of the study

Nearly one in twenty persons worldwide suffer from fibromyalgia (FM), which is the third most prevalent diagnosis in clinical rheumatology [10]. FM has a strong influence on psychological well-being and causes many psychological problems such as depression and anxiety. Among population, FM prevalence ranged from 0.2 to eight%. In the Eastern Mediterranean, the combined regional prevalence of FM is 4.43%. Schoolchildren, particular populations including adults with autoimmune rheumatic disorders, chronic hepatitis C infection and chronic depression have all been included in published research on the prevalence of FM in Egyptians [11].

In Egypt, fibromyalgia frequently coexists with autoimmune inflammatory diseases such as rheumatoid arthritis in older adults, women, and those who suffer from chronic depression [12]. Thus, patient counseling and education are considered an important component of multimodal therapy which can help patients with fibromyalgia to live with pain, reduce physical symptoms and improve psychological well-being.

## Methods

### Aim

The study aimed to evaluate the effect of counselling intervention on symptoms, pain acceptance, and the psychological well-being of patients with fibromyalgia. This aim was achieved through:

1. Assessing the patients’ knowledge regarding fibromyalgia.
2. Assessing the severity of symptoms among patients with fibromyalgia.
3. Assessing pain acceptance among patients with fibromyalgia.
4. Assessing the psychological well-being among patients with fibromyalgia.
5. Developing and implementing counselling interventions for patients with fibromyalgia.
6. Evaluating the effect of counselling intervention on symptoms, pain acceptance, and psychological well-being among patients with fibromyalgia.

#### Research hypothesis

1. Implementation of counseling intervention for patients with fibromyalgia will have a positive effect on symptoms management.
2. Implementation of counseling intervention for patients with fibromyalgia will have a positive effect on their pain acceptance.
3. Implementation of counseling intervention for patients with fibromyalgia will have a positive effect on their psychological wellness.

### Operational definitions

**Symptoms** are the patient’s physical symptoms associated with fibromyalgia including “pain – fatigue and energy, sleep problems, gastrointestinal tract problems and cognitive problems” [13].

**Pain acceptance** refers to the patient’s level of pain associated with fibromyalgia.

**Psychological wellbeing** refers to a patient’s level of anxiety, depression, positive well-being, self-control, vitality and general health [14].

### Research design

A quasi-experimental (study and control group) design was used to explore the effect of the counselling intervention on symptoms, pain acceptance and psychological well-being of patients with fibromyalgia.

### Study setting

The study was conducted in the outpatient rheumatology clinic El-Demerdash Hospital affiliated with Ain Shams University Hospitals, Cairo, Egypt. The clinic is on the ground floor, and it receives patients on Sunday and Wednesday weekly.

### Subject

A purposive sample of 120 adult patients confirmed with fibromyalgia diagnosis according to and meet the inclusion criteria.

## Inclusion criteria

- Adult patients (18–65 years of age) from both genders with confirmed primary fibromyalgia diagnosis no less than one year and receive the same medications.
- Patients who didn’t receive any counseling interventions about fibromyalgia and agreed to participate in the study.

## Exclusion criteria

- Female patients who were pregnant.
- Patients with physical and cognitive impairment.
- Patients with FM due to other causes of chronic pain such as presence of other autoimmune inflammatory diseases, parathyroid or thyroid gland disorder, metastatic cancer, chronic kidney or liver diseases, multiple myeloma or infection during the study.

### Sample size

A total population of 520 patients required a sample size of 120 to reach a confidence level of 90%. Therefore, using the statistical method outlined, the study enlisted 120 patients. These patients were randomly selected and categorized into two groups: a control group consisting of 60 patients who did not receive the counselling intervention, and a study group comprised of 60 patients who did receive the counselling intervention. The calculation for the sample size was performed by setting the power of the test at 80% and the confidence interval at 90%, with an acceptable margin of error adjusted to 5%, utilizing the following equation:

Type I error (α) is set at 0.05, while Type II error (β) is at 0.2. The power of the test is 0.80, and the equation is 120 = (520 × 0.50(1–0.50)/ [520–1 × (0.05 × x^2 ÷ 1.64 × x^2)] + 0.50(1–0.50). Here, n denotes the sample size, z is the standard score (1.64), and d refers to the error rate (0.05). p represents the proportion of availability and neutral ratio (0.50), and N indicates the population size.

In this context, p is the pooled proportion derived from prior research; d is the anticipated difference in the proportion of events; Zα is for a 5% significance level, and Zβ is 0.84 for an 80% study power.

### Data collection tools

The following five tools were used to collect data:

#### Tool I: structured interview questionnaire for patients with fibromyalgia

The researchers developed this tool based on recent and related literature. It included three parts as follows:

### Part 1: socio-demographic characteristics data of the patients

Eight questions on sex, age, marital status, occupation, monthly income, education, treatment costs, residence, and smoking habits made up this tool.

### Part 2: medical health history of the patients

It contained the following:

Current medical history (5 closed-ended and multiple-choice questions about height, weight, BMI, primary complaint, and duration since fibromyalgia diagnosis).

The history section included four multiple-choice questions and closed-ended questions about past hospital stays, surgeries, chronic illnesses, and non-disease-related drugs.

A single yes-or-no question about the family history of the same illness made up the family history section.

### Part 3: fibromyalgia knowledge assessment questionnaire

This tool was used to assess patients’ knowledge regarding fibromyalgia pre and post the counseling intervention implementation to identify their level of knowledge and how it has been improved after intervention to enhance their abilities to understand the disease and its correlated complications. Usually, pre-intervention assessments help in identifying the patients (informational) needs based on their level of knowledge. The researchers developed it based on the recent related literature [15–16].

### Scoring system for the patients’ knowledge

This tool had 20 statements divided into two sections for the purpose of rating patients’ knowledge. The answers were either yes or no; the right response received one score, while the incorrect response received zero. Before calculating the total score for all the knowledge surveys, the scores of each statement for each segment were added up to create a total score for each component [15–[16].

Based on statistical analysis, patients’ knowledge was divided into two categories: satisfactory and unacceptable.

- A score of ≥ 70% was considered satisfactory.
- A score of < 70% was considered unsatisfactory.

### Tool II: fibromyalgia symptoms questionnaire

This tool was used to examine symptoms currently experienced by the patients because of fibromyalgia and these symptoms were assessed for severity before and after counselling intervention. It has been developed and prepared by researchers based on the related literature [15–[16].

### Scoring system

It included questions regarding fibromyalgia-associated signs and symptoms which were 24 questions grouped into 4 sections (pain – fatigue and energy “had 12 questions”, sleep problems “had 4 questions”, gastrointestinal tract problems “had 5 questions” and cognitive problems “had 3 questions”. The symptoms were divided into four categories ranging from 1 = no time, 2 = a little of the time, 3 = some of the time score, and 4 = most of the time. High score indicated high severity symptoms.

### Tool III: chronic pain acceptance questionnaire (CPAQ)

It was adopted from [17], and is employed to measure acceptance of pain. The two main components of pain acceptance were evaluated using a self-report questionnaire and a 20-item descriptive scale: (1) Activity engagement (the extent to which the respondent is able to carry out everyday tasks in spite of discomfort), comprising items 1, 2, 3, 5, 6, 8, 9, 10, 12, 15, and 19 and (2) Pain willingness, which encompassed items 4, 7, 11, 13, 14, 16, 17, 18, and 20. Pain willingness is the capacity to feel pain without attempting to control, alter, or escape it.

### Scoring

A seven-point rating system, ranging from 0 (never true) to 6 (always true), was used to assess the 20 items on the CPAQ. The Pain Willingness Score (PWS) components 4, 7, 11, 13, 14, 16, 17, 18, and 20 are composed of nine items that have inverse scores. The Activity Engagement Score (AES) is composed of eleven things that are assessed directly: items 1, 2, 3, 5, 6, 8, 9, 10, 12, 15, 19. PWS and AES are added together to create the overall score (CPAQ), which has a range of 0 to 120. Higher engagement in activities and a greater acceptance of pain are reflected in higher scores. PWS and AES were added together to create the overall score (CPAQ), which was determined as follows:

- High function-High satisfaction = 100–120.
- High function - medium satisfaction = 80 – less than less than 100.
- Medium function - Low satisfaction = 60 - less than 80.
- Low function - medium satisfaction = 40 – less than 60.
- Low function - Low satisfaction = 20 – less than 40.
- Very low function - Very low satisfaction 0–20.

### Tool IV: psychological well-being scale

This tool has been adopted [18], and evaluated the patient’s degree of anxiety, depression, positive well-being, self-control, energy, and general health in order to determine psychological wellbeing.

### Scoring

This tool had eighteen items that measured subjective psychological well-being and distress. The time span of interest for all the items is the previous month. Reverse scoring applies to items 1, 3, 6, 7, 9, 11, 13, 15, and 16. Due to the reverse scoring of these elements, the overall score is reduced by 14 to produce a range of 0-110. “Severe distress,” “moderate distress,” and “positive well-being” are represented by total scores ranging from 0 to 60, 61 to 72, and 73 toward 110.

#### Preparatory phase

The following actions were taken to complete this phase:

1. Creating data gathering tools following a review of recent publications in the field, mainstream research, and other sources.
2. Using a thorough analysis of the literature and other resources, outlining every topic that should be included in the pamphlet and counseling intervention.
3. The process of creating the counseling intervention, including its design and content preparation.
4. Getting the advice of specialists to guarantee the legitimacy of the booklet.

### Tool validity and reliability

Nine experts—five from medical surgical nursing and four from psychiatric/mental health nursing—were asked to provide their thoughts on the tool’s format, layout, and knowledge correctness. Each tool’s Pearson correlation coefficient (r) and internal consistency (Cronbach alpha) were examined.

**Table:** 

Tool	Reliability “Pearsons	Cronbach’s Coefficient
Structured Interview Questionnaire for Patients with Fibromyalgia	0. 88	0.93
Fibromyalgia Symptoms Questionnaire	0. 85	0.82
Chronic pain acceptance questionnaire	0.82	0.889
Psychological well-being scale	0.89	0.92

#### Pilot study

A pilot study was carried out on (12 patients with fibromyalgia) 10% of the total sample before conducting the actual study to ensure clarity of the questions, applicability of data collection tools, and time needed to complete them. All subjects who were involved in the pilot study were excluded from the main study sample. The tool was finalized based on the results of the pilot study.

#### Fieldwork

The collection of data and application of counselling intervention lasted over a period of nine months; starting in June 2023 and ending in February 2024, through the following phases:

- Assessment and planning phase.
- The researchers visited the outpatient rheumatology clinic 2 days on Sunday and Wednesday during morning shifts (9.00 am to 2.00 pm).
- The researchers obtained the patients’ written consent for participating in this study after explaining the aim of the study.
- Filling in the previously mentioned tools was done by the researchers before implementation of the counseling.
- These tools were completed within an average time of 60 min.

#### Implementation phase

- The counseling sessions were conducted in the room at outpatient rheumatologic clinic.
- Total number of the sessions of counselling intervention was 5 sessions. Each session of them took one and half hours/ day for 2 days per week.
- Implementation of counselling intervention lasted over a period of 6 months for all patients.
- At the beginning of the first session an orientation of counseling intervention and its purpose took place. The importance and benefit of counseling intervention were explained to the patients to motivate them to follow counselling intervention, which included in it.
- The first counseling sessions included fibromyalgia information. The second counselling session included methods of fibromyalgia treatment. The third counselling session included instructions regarding controlling pain, managing fatigue, and performing physical exercises for muscle strengthen. The fourth counselling session included instructions regarding healthy nutrition, managing gastrointestinal problems, sleep hygiene. The fifth counselling session included instructions regarding cognitive improvement and controlling of psychological pressures.

The interventions focused on physical, educational, psychological, behavioral, or integrative awareness. Physical interventions involved appropriate nutrition, home care and complementary treatment at home, insomnia treatment, and how to adapt with FM. While psychological interventions focused on how to cope with stress and anxiety related to FM, listening to music, direct imagination, meditation, etc. On the other hand, behavioral or integrative interventions include learning how to solve problems, critical thinking, social interaction, etc. These interventions differ from existing multimodal therapy strategies, which focus on patient self-care interventions, while the treatment strategies depend on medication and surgical interventions. This intervention has been developed by researchers based on literature reviews.

#### Evaluation phase

It emphasized determining the effect of the counseling intervention on symptoms, pain acceptance, and psychological wellbeing among patients with fibromyalgia through control/study assessment using the previously mentioned tools, concerning knowledge, was assessed pre and immediately after implementation of the counseling intervention.

### Statistical analysis

The data was collected, coded and entered a suitable excel sheet. Data was analyzed using the statistical package for social sciences, version 20.0 (SPSS). The statistical analysis was done using percentage (%), mean, standard deviation; Chi-Square (x2) was used in order to compare proportions between two qualitative parameters, Spearman’s rank correlation coefficient (rs) was used to assess the degree of association between two sets of variables if one or both of them was skewed and the confidence interval was set to 95% and the margin of error accepted was set to 5%. The observed differences and association were considered as follows:

- Nonsignificant (NS) P ˃ 0.05.
- Significant (S) *P* ≤ 0.05.
- Highly Significant (HS) *P* ≤ 0.01.

## Results

Regarding the mean age of the study group was 36.34 ± 9.43, while the mean age of the control group was 34.34 ± 8.42. Regarding the gender of the study and control groups, it was found that 76.7% of the study group & 90.0% of the control group patients were females. Regarding the marital status of the patients under study, it was found that the percentages of the study and control groups who married were 50% and 90% respectively. As regards their educational level, 48.4% of the study group were primary education and 86.6% of the control group could read and write. As regards their occupational level, about 53.3% of the study group patients and 83% of the control group patients were not working.

Concerning the monthly income, 61.7% of the patients in the study group and 68.3% of control groups had insufficient monthly income. In relation to residence, this table shows that 78.3% of the study group and 91.4% of the control group resided in the urban areas. As well as the percentage of the patients in the study and control groups regarding their smoking status, it was found that 76.7% & 90% respectively were nonsmokers. See more in Table 1.

**Table 1:** Frequency distribution of patient knowledge about fibromyalgia throughout study phases (n = 120)

Items	Study Group (*n* = 60)		Control Group (*n* = 60)	
	No.	%	No.	%
**Age (Years):**				
20-> 30	18	30	13	21.7
30- > 40	36	60	34	56.6
40 years or more	6	10	13	21.7
**Mean** *±* **SD**	**(36.34 ± 9.43)**		**(34.34 ± 8.42)**	
**Sex**:				
Male	14	23.3	17	10
Female	46	76.7	43	90
**Marital Status**:				
Married	30	50	29	90
Single	19	31.7	18	5.7
Divorced /Separated	11	18.3	13	4.3
**Level of Education**:				
Read & Write	12	20	18	68.6
Primary Education	29	48.4	22	18.6
secondary Education	11	18.3	11	10
University education	8	13.3	9	2.8
**Occupational Level**:				
Working	28	46.7	23	17
Not working	32	53.3	37	83
**Monthly Income**:				
Sufficient	23	38.3	19	31.7
Insufficient	37	61.7	41	68.3
**Residence**				
Urban	47	78.3	39	91.4
Rural	13	21.7	21	8.6
**Smoking status**				
**Yes (men)**	14	23.3	17	10
**No (women)**	46	76.7	43	90

According to the knowledge of patients under study about fibromyalgia there was a statistically significant difference in the study group’s total satisfactory level of knowledge about fibromyalgia during the two phases of the study. See more in Table 2.

**Table 2:** Comparison of total mean score regarding physical symptoms among study and control group throughout study phases (*n* = 60)

Item	Pre		Post		Test		S
	Mean	*±*SD	Mean	*±*SD	T	*P*	
Definition of fibromyalgia	1.5	1.2	3.1	1.6	4.8	< 0.01	HS
Causes of fibromyalgia	2.4	1.7	4.0	1.7	4.2	< 0.01	HS
Risk factors of fibromyalgia	1.2	1.0	3.6	1.1	9.6	< 0.01	HS
Signs /Symptoms of Fibromyalgia	2.9	0.7	3.4	1.4	2.0	> 0.05	S
Methods of treatment	1.4	0.7	2.6	1.1	6.1	< 0.01	HS
How to deal with signs /symptoms of fibromyalgia	1.6	1.0	2.4	1.7	2.9	< 0.01	HS
How to deal with psychological symptoms	0.9	0.9	2.4	0.9	7.2	< 0.01	HS
Total	14.7	5.1	25.3	9.5	6.2	< 0.01	HS

Related to the comparison between the study and control groups regarding fibromyalgia physical symptoms. Mean Score before and after counseling intervention Implementation, Table 3 illustrates that the patients in the study group had statistically significant difference post counseling intervention implementation regarding pain, fatigue and energy problems, sleep disturbances, headache, gastrointestinal disturbances, and cognitive problems at p-value were < 0.001. While patients in the control group had a non-significant difference post counseling intervention implementation regarding pain, fatigue and energy problems, sleep disturbances, headache, gastrointestinal disturbances, cognitive problems (0.394,0.254, 0.099, 0.704, 0.117, respectively).

**Table 3:** Comparison between the study and control groups regarding fibromyalgia physical symptoms. Mean score before and after counseling intervention implementation (*n* = 120)

Physical symptoms	Patients with fibromyalgia (*N* = 60) Study group				t-test	*P*-value	Patients with fibromyalgia (*N* = 60) Control group				t-test	*P*-value
	Pre- Counseling		Post Counseling				Pre- Counseling		Post Counseling			
	Mean	SD	Mean	SD			Mean	SD	Mean	SD		
Pain – fatigue and energy problems	15.4	4.45	25.07	4.03	23.15	0.01**	16.2	3.34	17.7	2.11	0.159	0.394
Sleep disturbances	7. 2	2.1	11.3	3.54	9.34	0.01**	6. 9	2.6	7. 1	1.9	0.622	0.254
Gastrointestinal disturbances	10.2	0.57	15.6	3.01	11.23	0.01**	11.2	0.09	10.4	1.89	1.901	0.704
Cognitive problems	5.4	1.31	9.43	2.56	7.45	0.01**	6.8	2.11	8.3	1.78	1.003	0.117

**Fig. 1:**
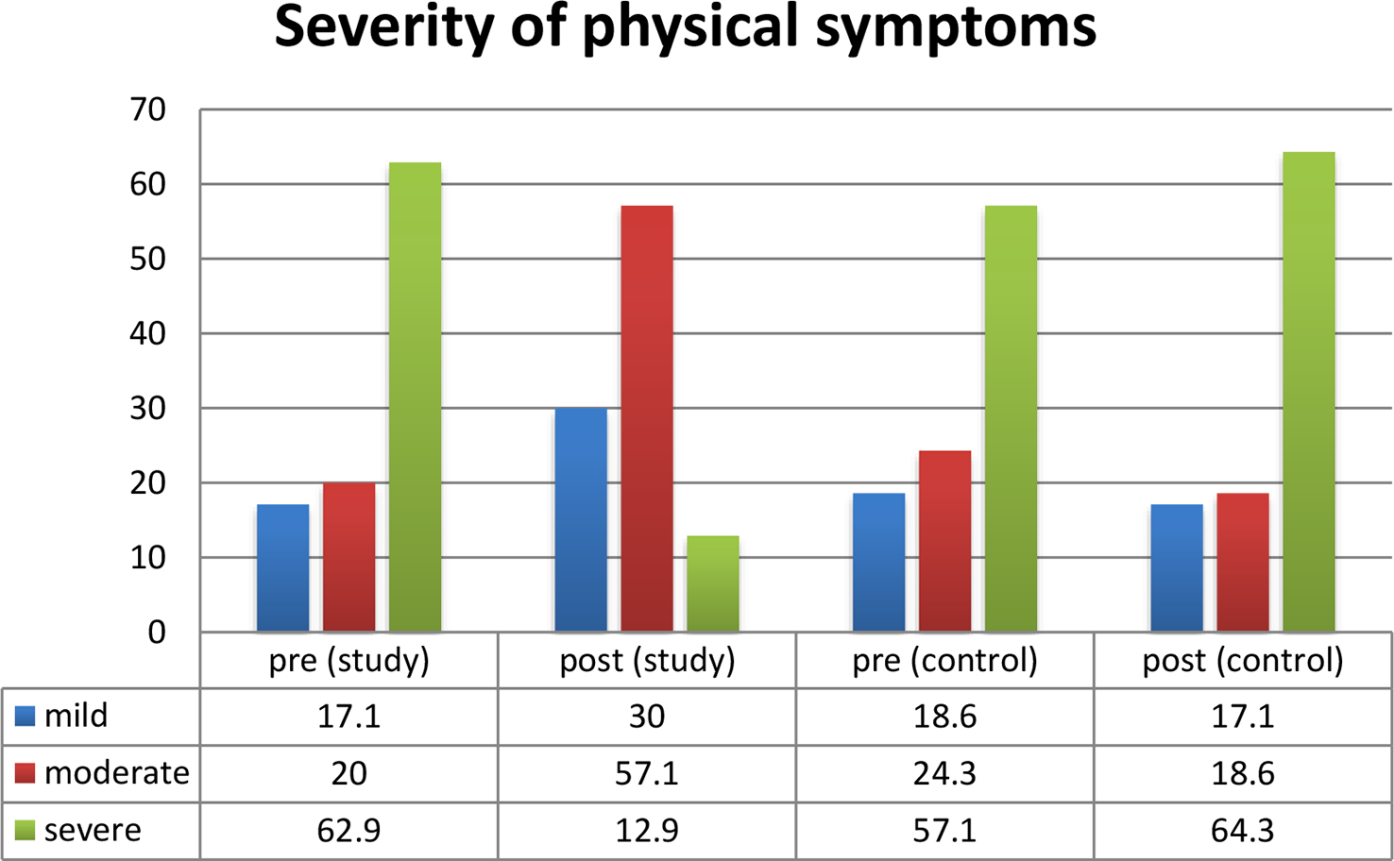
Total level of physical well-being pre and post program implementation (*n* = 120)

Figure 1 describes that there was a statistically significant difference between the study and control groups regarding total level of physical symptoms post implementation of counseling intervention for the study group.

Regarding the comparison between the study and control groups regarding pain acceptance scale. Mean Score before and after counseling intervention Implementation, Table 4 clarified that the patients in the study group had statistically significant difference post counseling intervention implementation regarding Anxiety, Depression, Positive well-being, Vitality, General Health (0.03*, 0.01**, patients in the study group had statistically significant difference post counseling intervention implementation regarding Pain willingness subscale, Activity engagement subscale at p-value was < 0.001. While patients in the control group had a non-Significant difference post counseling intervention implementation regarding Pain willingness subscale, Activity engagement subscale (0.161, 0.327, respectively).

**Table 4:** Comparison between the study and control groups regarding pain acceptance scale. Mean score before and after counseling intervention implementation (*n* = 120)

Pain acceptance scale	Study group				t test	*p*-value	Control group				t test	*p*-value
	Pre-Program		Post Program				Pre-Program		Post Program			
	Mean	SD	Mean	SD			Mean	SD	Mean	SD		
Pain willingness subscale	5.7	2.12	13.6	4.74	3.645	0.01**	4.9	3.45	5.8	2.45	1.44	0.161
Activity engagement subscale	6.2	2.13	15.5	4.76	3.988	0.01**	7.4	1.99	7.1	2.13	1.99	0.327
Total level of pain acceptance	11.9	4.25	29.1	9.5	3.189	0.01**	12.3	5.44	12.9	4.58	1.80	0.083

(*) Statistically significant at *p* < 0.05, (**) Statistically highly significant at *p* < 0.001, non-significant at *p* < 0.05

**Fig. 2:**
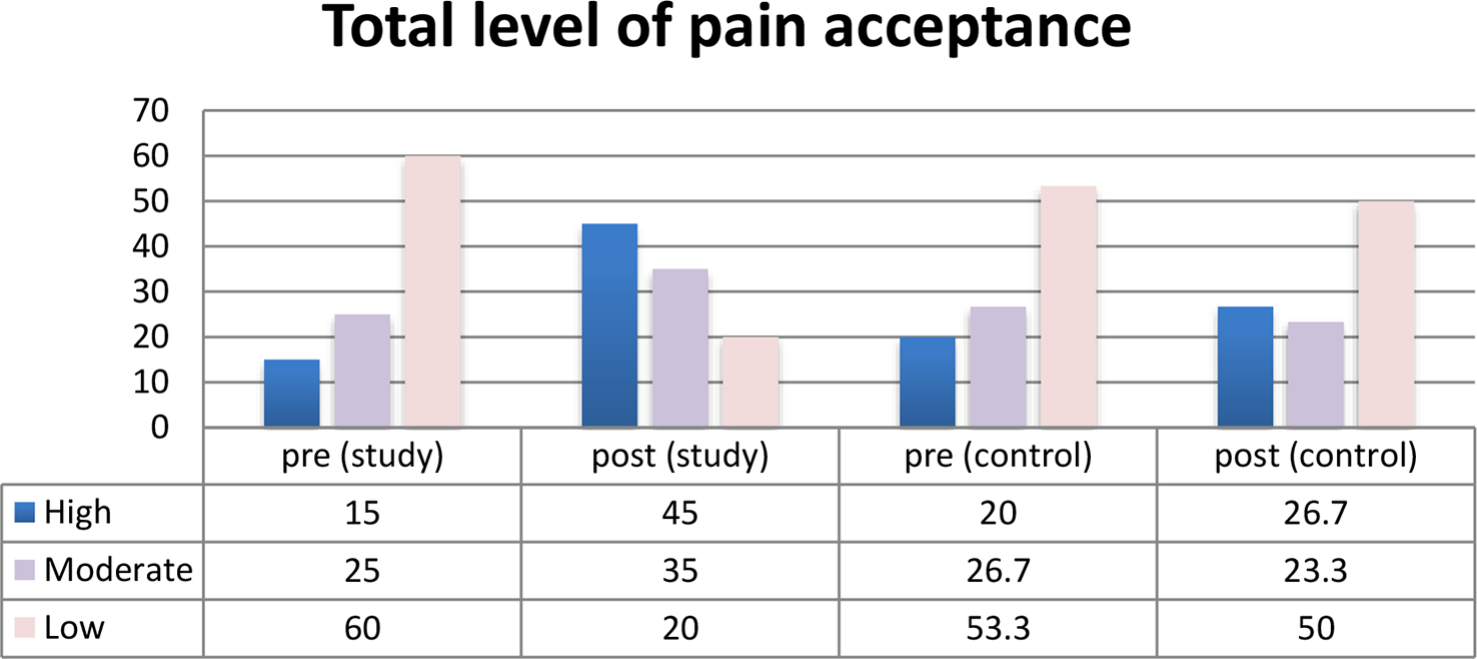
Total level of pain acceptance among patients before and after counseling intervention Implementation (*N* = 120)

Figure 2 describes that there was statistically significant difference between the study and control groups regarding total level of pain acceptance post implementation of counseling intervention for the study group.

Related to the comparison between the study and control groups regarding psychological wellbeing. Mean Score Pre- and Post-Program Implementation, Table 5 clarified that the patients in the study group had statistically significant difference post counseling intervention implementation regarding Anxiety, Depression, Positive well-being, Vitality, General Health (0.03*, 0.01**, 0.01**, 0.01**, 0.01**, respectively). While patients in the control group had a non-significant difference post counseling intervention implementation regarding Anxiety, Depression, Positive well-being, Self-control, Vitality (0.083, 0.159, 0.322, 0.327, respectively).

**Table 5:** Comparison between the study and control groups regarding psychological wellbeing. Mean score Pre- and Post-Program implementation (*N* = 60)

Psychological wellbeing	Study group				T-test	*P*-value	Control group				t-test	*P*-value
	Pre-Program		Post Program				Pre-Program		Post Program			
	Mean	SD	Mean	SD			Mean	SD	Mean	SD		
Anxiety	5.3	1.63	8.9	2.76	7.34	0.03*	4.9	1.43	5.6	2.13	1.76	0.083
Depression	4.2	3.61	8.6	3.87	7.41	0.01**	4.8	2.91	4.1	3.43	1.92	0.159
Positive well-being	4.6	2.57	9.43	2.67	11.56	0.01**	5.1	3.13	4.9	2.98	1.49	0.159
Self-control	4.4	3.54	6.1	4.12	13.45	0.14	4.7	3.87	5.2	1.99	1.35	0.322
Vitality	3.4	3.2	9.34	2.32	21.22	0.01**	3.9	1.97	3.7	2.31	1.65	0.327
General Health	2.6	1.53	4.21	3.42	45.21	0.01**	3.9	2.11	2.6	1.56	1.45	/

(*) Statistically significant at *p* < 0.05, (**) Statistically highly significant at *p* < 0.001, non-significant at *p* < 0.05

Regarding correlation coefficient of the study and control groups regarding the total level of Pain acceptance, Physical wellbeing and psychological wellbeing before and after implementation of counseling intervention, Table 6 clarifies that the patients in the study group had statistically significant difference post counseling intervention implementation.

**Table 6:** Correlation coefficient the study and control groups regarding the total level of pain acceptance, physical wellbeing and psychological wellbeing before and after implementation of counseling intervention

Study variables		Post-intervention		
		Pain acceptance	Physical symptoms	Psychological wellbeing
Post- intervention	Physical symptoms		0.312**	0.512**
	Pain acceptance	0.312**		0.631*
	Psychological wellbeing	0.512**	0.631*	

## Discussion

Fibromyalgia (FM) is a disorder characterized by widespread pain that negatively impacts physical and psychological well-being. Counselling interventions may help them to accept the pain and improve their physical and psychological well-being. nurses usually support patients with fibromyalgia from diagnosis to treatment and management of their conditions and improving their quality of life [8].

Regarding the sociodemographic data, the present study revealed that the Mean + SD of age study group was 36.34 ± 9.43, while the control group was 34.34 ± 8.42, and regarding to the sex, more than three quarters of both groups were females and not smokers. Add to that, nearly two thirds of the study group’s monthly incomes are insufficient while more than three quarters of the control group were sufficient. These results are inconsistent with [19] in their study entitled; Effectiveness of group acceptance and commitment therapy for fibromyalgia: A 6-month randomized controlled trial (EFFIGACT study), which stated that most patients were older than 65 years and a group ACT intervention produces a greater increase in global functional status than recommended medications and no treatment. From the researcher’s point of view, most cases now are suffering from joints problems and during the aging progress. So, they usually have joints and muscles pain, and it is important to assess the patients’ sociodemographic characteristics. Also, [20] stated that usually the low educational level, high age, and low socioeconomic status as associative factors for fibromyalgia, and depression have been a common comorbidity as well as associated with a higher impact of the disease.

Regarding the patient’s knowledge about fibromyalgia of the study group, the study showed that there were a Statistically highly significant at *p* < 0.001 pre and post regarding the concept, causes, how to deal with signs and symptoms and risk factors of fibromyalgia and statistically highly significant at *p* < 0.001of total knowledge pre and post. This is related to [8], who mentioned that the positive effect of acceptance remains significant also when adjusting for potentially relevant factors and by increasing patients’ knowledge regarding the disease, risk factors, and treatment.

While patient’s knowledge about fibromyalgia of the control group the study showed that there was a non-Significant at *p* < 0.05 pre and post regarding causes, risk factors, and how to deal with signs /symptoms of fibromyalgia and non-Significant at *p* < 0.05 pre and post of total knowledge pre and post. This results in line with [21] who showed that about 10% had a low knowledge, nearly half medium and less than one half had a high level of knowledge. Furthermore, there was no correlation between FM patients’ knowledge of physical exercise and their physical activity habits.

According to the researcher’s point of view, after getting instruction and information regarding the illness and its treatment, patients’ knowledge grows. Because patients’ knowledge and abilities have improved, there are typically disparities between their pre- and post-educational sessions regarding the disease. This relates to [22], who said that patient education is recommended as the initial treatment for fibromyalgia. It is defined as a sequence of educational activities planned by professionals with the intention of improving a patient’s health by changing the patient’s perspective on an illness. In this way, patient education can reassure a patient regarding the gravity, validity, and outlook of their illness.

Regarding the physical wellbeing The results of the current study are consistent with a study by [23] titled “Efficacy of rehabilitation with Tai Ji Quan in an Italian cohort of patients with Fibromyalgia Syndrome,” which found that patients in the experimental group significantly improved Physical Index, Physical activity, physical role, bodily pain, general health, vitality, sleep duration, and sleep disturbance, while patients in the control group did not improve in any of these parameters. The study also found a highly statistically significant difference between the study and control groups in terms of pain, fatigue and energy issues, sleep disturbances, headaches, gastrointestinal disturbances, and cognitive issues following the implementation of counseling intervention. A study by [10] titled “A psychoeducational intervention is a treatment for fibromyalgia syndrome” also supports this conclusion, stating that the studies showed improvements in physical symptoms such stiffness, exhaustion, and sleep quality. According to the study, bodily problems including sleep, accepting pain, and managing exhaustion typically get better when the patient’s counseling and knowledge are increased through informational sessions or educational programs.

In relation to psychological wellbeing This study’s findings regarding psychological wellbeing revealed a highly statistically significant difference between the study and control groups regarding depression, positive well-being, and general health following the implementation of counseling intervention., this is consistent with the study by Gómez [24] titled “Psychoeducation for Patients with Fibromyalgia: A Systematic Review” found that psychoeducational programs were effective in improving general health related quality of life and reducing negative moods among patients with fibromyalgia due to overlapping symptoms between the two conditions [25]. A study by [26]) titled “Effects of Manual Therapy on Fatigue, Pain, and Psychological Aspects in Women with Fibromyalgia” further supports this conclusion, demonstrating that manual therapy reduced tension-anxiety levels in fibromyalgia patients. Depression is typically a prevalent comorbid disorder in FMS and has a significant role in disability and low quality of life. However, because fibromyalgia and depression share symptoms, it can be challenging to diagnose depression and anxiety in fibromyalgia patients [25].

According to the researcher’s point of view, most patients’ psychological issues would be resolved if they receive support and emotional or psychological awareness, particularly for those with chronic illnesses. This relates to [27], who claimed that people with fibromyalgia (FM) have poorer cognitive function, reduced physical activity, and more severe depression symptoms. There is mounting evidence that frequent participation in physically demanding activities enhances psychosocial functioning in people with FM, even though exercise therapy and physical activity are typically excluded from the traditional treatment for this illness. The impact of Zumba dancing and aerobic fitness training, two different forms of therapy, on depressive symptoms, motor function, and working memory in female FM patients. The significance of psychological interventions for patients with FM was also mentioned by [28]. When patients have mild impairment, internet psychological interventions can be the first line of treatment. If patients do not respond or clinical severity increases, more intensive and demanding interventions (like group or individual therapy) can be used.

Regarding the level of pain acceptance, this study’s findings are consistent with a study by [26], which found that applying manual therapy to fibromyalgia patients with moderate pressure for 15 min on the posterior cervical musculature reduced their perception of pain. The study also found a highly statistically significant difference between the study and control groups regarding the Pain willingness subscale and the Activity engagement subscale following the counseling intervention. Furthermore, [29] discovered that all fibromyalgia patients need to receive instruction in self-directed physical exercise, and that pain psychology techniques like behavioral, cognitive, or mindfulness-based stress reduction ought to be suggested for patients, all of whom are subject to periodic awareness and nursing interventions. According to the study, following an exercise program or activities on occasion usually lowers the amount of pain, especially after following advice on how to exercise often in all areas of pain, such as joint or muscle discomfort.

According to [28] advice for patients with FM, nonpharmacologic interventions such as psychotherapy, exercise, dietary modifications, and patient education have the potential to improve overall quality of life and reduce pain. This should begin early in the course of the sickness. Cognitive/Psychological Therapy (CBT) has been shown to be the most successful psychological treatment for fibromyalgia patients, helping them with their pain levels, depression, and sleep problems. Exercise, including aerobic and anaerobic exercise, aquatic therapies, Qi Gong therapy, and Tai Chi, has demonstrated promise in lowering pain and enhancing function, however the long-term consequences are still being studied. Supplements, dietary changes, adjunct therapies including acupuncture and transcutaneous electric stimulation, and other treatments may help improve management. Shady et al. [30] stated that the impact of effective nursing practices and interventions on the patient’s standard of care, with a particular emphasis on the significance of nursing program interventions on the improvement of patients’ well-being, which is founded on ongoing monitoring and nurse-patient education.

## Conclusion

Patients with fibromyalgia under research report statistically significant improvements in their psychological well-being, pain acceptance, and symptoms when counseling interventions are used.

### Recommendations

- In all fibromyalgia treatment clinics, counseling interventions ought to be included in the standard nursing care provided to those patients.
- Full-day therapy sessions that offer the most recent details on fibromyalgia and its management strategy.
- Provide fibromyalgia patients with follow-up care by phone, online, and in-person visits from qualified nurses.

### Limitations of the study

This study included a number of limitations, such as the potential for bias caused by the use of self-reported data for nursing knowledge and practices, which could have affected implementation and results. Because there was no randomization in the quasi-experimental design, it is difficult to draw conclusions about causality, and baseline variations between the study and control groups could have skewed the results. Furthermore, there is potential for more research into patient perspectives on care given the study’s scant examination of patient experiences beyond satisfaction ratings.

## Data Availability

No datasets were generated or analysed during the current study.
